# Immunomodulatory Properties of Mesenchymal Stromal Cells: An Update

**DOI:** 10.3389/fcell.2021.637725

**Published:** 2021-02-09

**Authors:** Luise Müller, Antje Tunger, Manja Wobus, Malte von Bonin, Russell Towers, Martin Bornhäuser, Francesco Dazzi, Rebekka Wehner, Marc Schmitz

**Affiliations:** ^1^Institute of Immunology, Faculty of Medicine Carl Gustav Carus, TU Dresden, Dresden, Germany; ^2^National Center for Tumor Diseases (NCT), Partner Site Dresden, Dresden, Germany; ^3^Department of Medicine I, University Hospital Carl Gustav Carus, TU Dresden, Dresden, Germany; ^4^German Cancer Consortium (DKTK), Partner Site Dresden, and German Cancer Research Center (DKFZ), Heidelberg, Germany; ^5^Center for Regenerative Therapies Dresden (CRTD), TU Dresden, Dresden, Germany; ^6^School of Cancer and Pharmacological Sciences and KHP Cancer Research UK Centre, King’s College London, London, United Kingdom

**Keywords:** mesenchymal stromal cells, immunomodulation, macrophages, dendritic cells, T cells

## Abstract

Mesenchymal stromal cells (MSCs) are characterized by an extraordinary capacity to modulate the phenotype and functional properties of various immune cells that play an essential role in the pathogenesis of inflammatory disorders. Thus, MSCs efficiently impair the phagocytic and antigen-presenting capacity of monocytes/macrophages and promote the expression of immunosuppressive molecules such as interleukin (IL)-10 and programmed cell death 1 ligand 1 by these cells. They also effectively inhibit the maturation of dendritic cells and their ability to produce proinflammatory cytokines and to stimulate potent T-cell responses. Furthermore, MSCs inhibit the generation and proinflammatory properties of CD4^+^ T helper (Th)1 and Th17 cells, while they promote the proliferation of regulatory T cells and their inhibitory capabilities. MSCs also impair the expansion, cytokine secretion, and cytotoxic activity of proinflammatory CD8^+^ T cells. Moreover, MSCs inhibit the differentiation, proliferation, and antibody secretion of B cells, and foster the generation of IL-10-producing regulatory B cells. Various cell membrane-associated and soluble molecules essentially contribute to these MSC-mediated effects on important cellular components of innate and adaptive immunity. Due to their immunosuppressive properties, MSCs have emerged as promising tools for the treatment of inflammatory disorders such as acute graft-versus-host disease, graft rejection in patients undergoing organ/cell transplantation, and autoimmune diseases.

## Introduction

Mesenchymal stromal cells (MSCs) are characterized *in vitro* by the adherence to plastic surfaces, the expression of CD73, CD90, and CD105, the lack of expression of the hematopoietic and endothelial markers CD11b, CD14, CD19, CD34, CD45, CD79a, and detectable amounts of human leukocyte antigen (HLA)-DR as well as the capability to differentiate into adipocytes, chondrocytes, and osteoblasts (Dominici et al., 2006; Viswanathan et al., 2019). Additionally, the MSC committee of the International Society for Cell and Gene Therapy recommends to support the phenotypical characterization by functional assays confirming hallmark properties of MSCs such as secretion of soluble factors and immunomodulation (Viswanathan et al., 2019). While MSCs were first isolated from bone marrow (BM), a variety of tissues were found to harbor MSCs comprising adipose, placenta, umbilical cord (UC), dental pulp, and other tissues. Due to their accessibility, adipose tissue-derived (AD)-MSCs and UC-MSCs have gained an increasing popularity, especially for clinical studies. Even though all MSCs share certain characteristics, the discrimination between MSCs of different origins became particularly important since several studies have found striking differences, regarding not only their marker expression and cytokine profile but also their functional properties (Kozlowska et al., 2019; Ritter et al., 2019; Ménard et al., 2020; Petrenko et al., 2020; Song et al., 2020). For example, BM-MSCs secreted the highest amount of pro-angiogenic interleukin (IL)-8 and vascular endothelial growth factor compared to MSCs derived from adipose tissue, skeletal muscle, and skin, while AD-MSCs displayed the strongest ability to secrete IL-6 (Kozlowska et al., 2019). Additionally, BM-MSCs were found to have the most prominent immunosuppressive capacities in both cell contact-dependent and paracrine settings (Petrenko et al., 2020). Despite several challenges, MSCs have gained increasing attention in recent years. Their differentiation capability allows for their therapeutic use in regenerative medicine and tissue engineering. In addition, MSCs exhibit a low immunogenicity and display an extraordinary capacity to modulate immune responses. While these traits make MSCs attractive candidates for the treatment of immune-related disorders like autoimmune diseases, acute graft-versus-host disease (aGvHD), and sepsis, their modulatory action strongly depends on the environmental stimuli (Wang et al., 2016). It has been shown that under certain conditions, MSCs can promote immune responses by secreting proinflammatory cytokines and acting as antigen-presenting cells. Their immunostimulatory capabilities can be converted into an immunosuppressive phenotype by a process termed “licensing.” This phenotypic and functional shift is mediated by inflammatory cytokines such as interferon (IFN)-γ or tumor necrosis factor (TNF)-α (Krampera, 2011). The dual role of MSCs should be considered when assessing their immunomodulatory capacities and their use in clinical applications (Carvalho et al., 2019). Here, we focus on recent studies exploring the impact of MSCs on the phenotype and functional properties of monocytes/macrophages, dendritic cells (DCs), T cells, and B cells that play a major role in various immune-driven disorders. Furthermore, the cell membrane-associated and soluble molecules that contribute to the immunomodulatory effects of MSCs are summarized.

## Modulation of Innate Immunity by MSCs

### Monocytes/Macrophages

Macrophages are important components of innate immunity and play an important role in the pathogenesis of various immune-mediated diseases. Based on their phenotype and functional properties, macrophages can be classified into proinflammatory M1 and anti-inflammatory M2 macrophages (Shapouri-Moghaddam et al., 2018). Recently, it has been demonstrated that MSCs efficiently promote macrophage polarization toward the M2 type, which is considered to be beneficial in immune-driven disorders. This M2 polarizing effect of MSCs is mediated by various soluble molecules including prostaglandin E2 (PGE2) (Németh et al., 2009; Vasandan et al., 2016), indolamin-2,3-dioxygenase (IDO) (François et al., 2012), IL-6, hepatocyte growth factor (HGF) (Deng et al., 2016), IL-1 receptor antagonist (IL-1RA) (Luz-Crawford et al., 2016), tumor necrosis factor-inducible gene 6 protein (TSG6) (Ko et al., 2016), and transforming growth factor (TGF)-β (Liu et al., 2019a). Several studies suggest a key role of PGE2, as the inhibition of PGE2 or the cyclooxygenase-2 (COX2) pathway abrogated the observed inhibitory effects (Németh et al., 2009; Jin et al., 2019; Ortiz-Virumbrales et al., 2020). Previous studies using AD- or BM-MSCs indicated that the paracrine action is partially mediated by exosomes (Biswas et al., 2019; Cho et al., 2019; He et al., 2019; Liu et al., 2019b; Wang et al., 2020b). The exosomes contained TGF-β, C1q, semaphorins, and micro (mi) RNAs, regulating the macrophage polarization and inducing overexpression of programmed cell death 1 ligand 1 (PD-L1). Moreover, MSCs can alter the macrophage phenotype by manipulating their metabolic properties, such as glycolysis (Vasandan et al., 2016; Deng et al., 2020).

The MSC-induced M2 polarization is accompanied by an increased secretion of anti-inflammatory IL-10 and Arginase-1 and a reduced production of proinflammatory cytokines like TNF-α, IL-12, and IL-1β (Kim and Hematti, 2009; Yang et al., 2020). Additionally, exosomes from UC-MSCs reduced the expression of the NLR family pyrin domain containing 3 (NLRP3) inflammasome and involved downstream factors like caspase-1, IL-1β, and IL-6 in lipopolysaccharide (LPS)-stimulated macrophages (Jiang et al., 2019). Another study revealed that the effect of IL-10 production by murine MSC-primed macrophages is particularly important, as the depletion of IL-10 by antibodies abrogated beneficial effects of MSC treatment in a model of sepsis (Németh et al., 2009). M2 macrophages, characterized by reduced expression of costimulatory molecules and elevated secretion of anti-inflammatory cytokines like TGF-β, profoundly inhibit T cell responses and induce regulatory T cells (Tregs) (Savage et al., 2008; Schmidt et al., 2016), leading to further immunosuppression and supporting the positive effects of MSC therapy. MSCs also impair both the differentiation and effector function of monocytes. Maqbool et al. (2020) discovered a reduced expression of HLA-DR/DP/DQ and CD86 by monocytes and macrophages upon co-culture with human UC-MSCs. Functional analysis of this interaction revealed a significantly decreased phagocytic capacity and antigen-presenting ability of monocytes and macrophages when co-cultured with UC-MSCs. Furthermore, we investigated the impact of MSCs on various immunomodulatory properties of 6-sulfo LacNAc monocytes (slanMo), representing a subset of pro-inflammatory CD14^–^CD16^+^ non-classical monocytes, which may contribute to the pathogenesis of various inflammatory diseases (Ahmad et al., 2019). We found that MSCs profoundly suppress the capacity of slanMo to secrete TNF-α, IL-6, and IL-12, improve their IL-10 production, and efficiently inhibit the slanMo-induced proliferation of CD4^+^ and CD8^+^ T cells (Wehner et al., 2009). Additionally, de Witte et al. (2018) demonstrated that upon their phagocytosis by monocytes, UC-MSCs induce a CD14^++^CD16^+^CD206^+^ phenotype accompanied by increased IL-10 secretion and PD-L1 expression in mice. Further studies revealed that the phagocytosis of cord tissue (CT)-derived-MSCs is mediated by lipoprotein receptor-related proteins on monocytes and macrophages (Min et al., 2020). Moreover, the macrophage reprogramming upon phagocytosis of CT-MSCs was shown to be dependent on cytoplasmic RNA processing bodies (p-bodies), as p-body-deficient MSCs failed to suppress inflammation. P-bodies are membrane-less organelles that contain RNA, miRNA, and proteins which may facilitate macrophage polarization upon phagocytosis of MSCs (Kulkarni et al., 2010). We have discovered similar mechanisms, in which the immunosuppressive effects of MSCs depend on their apoptosis induced by recipient cytotoxic immune effector cells (Galleu et al., 2017; Cheung and Dazzi, 2018). Remarkably, the observed cytotoxicity differed significantly between clinical responders and non-responders to MSC therapy. Subsequently, apoptotic MSCs were shown to induce IDO production by recipient phagocytic cells in a GvHD mouse model and both the depletion of phagocytes and blockade of IDO reduced the beneficial effects.

### Dendritic Cells

Activated DCs display a unique capacity to induce T-cell responses and are the main producers of proinflammatory cytokines (Steinman and Banchereau, 2007; Qian and Cao, 2018). Due to these functional properties, DCs can essentially contribute to the immunopathogenesis of various disorders like aGvHD and autoimmune diseases. Their crucial role makes DCs attractive targets for immune-modulating therapies (Ganguly et al., 2013; Obregon et al., 2017). MSCs can efficiently inhibit the differentiation of DCs from hematopoietic stem cells and monocytes (Nauta et al., 2006; Ramasamy et al., 2007). The latter is facilitated by the downregulation of Cyclin D2, hindering monocytes from entering the G1 phase of the cell cycle. Additionally, MSCs impair the maturation of DCs, reducing their capacity to activate T cells. This is accompanied by a decreased expression of HLA-DR, CD40, OX40L, CD80, CD83, and CD86 (Jiang et al., 2005; Dong et al., 2018), as well as an increased PD-L1 expression (Lu et al., 2020). Moreover, MSCs shift the cytokine profile of DCs from proinflammatory toward immunoregulatory. For example, a decreased secretion of proinflammatory cytokines by CD1c^+^ DCs and an enhanced expression of IL-10 secretion by plasmacytoid (p)DCs upon co-culture with MSCs have been reported (Aggarwal and Pittenger, 2005). Similarly, the production of IL-12 by monocyte-derived DCs (moDCs) and IFN-α by pDCs was impaired when co-cultured with BM- and UC-MSCs, respectively (Jiang et al., 2005; Chen et al., 2019). These effects were mediated by PGE2, TSG6, IL-6, and macrophage colony-stimulating factor (M-CSF) as the inhibition of these molecules abolished the observed effects (Aggarwal and Pittenger, 2005; Jiang et al., 2005; Liu et al., 2014). Furthermore, murine AD-MSC-derived exosomes inhibited the IL-6 secretion and improved the release of IL-10 and TGF-β by DCs (Shahir et al., 2020).

MSCs promote the generation of tolerogenic DCs through different mechanisms. In particular, the induction of regulatory DCs seems to be dependent on Notch signaling (Li et al., 2008). The interaction between Jagged1 on BM-MSCs and Notch2 on DCs results in the generation of regulatory DCs (Li et al., 2020b). Furthermore, the MSC-mediated reduction of major histocompatibility complex (MHC) class II molecules, CD86, and CD40 could be mimicked by recombinant Jagged2 and blocked by a Notch inhibitor in a mouse model of acute lung injury (Lu et al., 2020). Besides cell contact-dependent mechanisms, the MSC-mediated generation of regulatory DCs is facilitated by soluble factors. For example, it has been shown that murine BM-MSCs induce the differentiation into regulatory DCs by HGF secretion (Lu et al., 2019). Further functional data revealed that rat MSCs inhibit the maturation of CD103^+^ DCs, leading to a decreased ability to prime CD8^+^ T cells (Zhang et al., 2020). Additionally, murine DCs that were treated with MSC-derived exosomes failed to stimulate T-cell proliferation upon LPS activation (Shahir et al., 2020).

## Regulation of Adaptive Immunity by MSCs

### T Cells

CD4^+^ T helper (Th) cells and CD8^+^ cytotoxic T cells (CTLs) play a pivotal role in the immunopathogenesis of aGVHD, autoimmune diseases, and other inflammatory disorders. Activated CTLs efficiently destroy cells and secrete large amounts of proinflammatory cytokines such as TNF-α and IFN-γ. Stimulated CD4^+^ T cells improve the capacity of DCs to induce CTLs by the interaction between CD40 on DCs and CD40 ligand on CD4^+^ T lymphocytes. Furthermore, CD4^+^ T cells provide help for the maintenance and expansion of CTLs by secreting cytokines such as IL-2. Previous studies have demonstrated that MSCs can profoundly suppress the proliferation of CD4^+^ and CD8^+^ T cells by both paracrine and cell contact-dependent mechanisms (Di Nicola et al., 2002; Krampera et al., 2003, 2006). While some authors argue that the anti-proliferative effect is not mediated by apoptosis (Krampera et al., 2006; Benvenuto et al., 2007), others reported an increased apoptosis of lymphocytes upon co-culture with MSCs (Zhao et al., 2012). This effect was dependent on the expression of Fas ligand (FasL) as the knock-down of FasL in MSCs by small interfering (si) RNA abrogated the effect on T lymphocytes as well as their therapeutic impact. As demonstrated by Akiyama et al. (2012), BM-MSCs secrete monocyte chemotactic protein 1 (MCP-1) to recruit T cells for FasL-mediated apoptosis in a mouse model of systemic sclerosis. More recently, it has been reported that particularly exosomes from murine MSCs mediate the cell cycle arrest of T cells through upregulation of cyclin-dependent kinase inhibitor 1B (CDKN1B) and downregulation of cyclin-dependent kinase 2 (Cdk2) (Lee et al., 2020). Besides the effect on the viability and proliferation of T cells, MSCs can also inhibit the generation and function of Th1 and Th17 cells while promoting Th2 cells and Tregs. BM-MSC-educated DCs mediate a shift from Th1 to Th2 and induce Tregs, which is accompanied by a decreased secretion of proinflammatory cytokines like IFN-γ, IL-17, and IL-6, whereas the production of IL-4 and IL-10 is increased (Wang et al., 2008; Ge et al., 2010). This effect seems to be partially dependent on IDO, as the impact was less prominent in IDO knock-out MSCs. Novel findings indicated that the inhibition of Th17 cells by murine MSCs is also mediated by HGF (Chen et al., 2020). Furthermore, it has been reported that BM-MSCs impair the IL-17 production by Th17 cells in a contact-dependent manner and induce their interconversion into Tregs (Luz-Crawford et al., 2019). They also provided evidence for a novel mechanism, in which MSCs transfer their mitochondria to Th17 cells, and subsequent experiments demonstrated that the artificial transfer of mitochondria impaired their IL-17 production. These findings are supported by another study that showed a mitochondrial transfer from MSCs to CD4^+^ rather than CD8^+^ T cells, which induced increased mRNA levels of FOXP3, CD25, cytotoxic T lymphocyte antigen 4 (CTLA-4), and TGFβ1 (Court et al., 2020). This subsequently led to the generation of a highly suppressive CD25^+^FoxP3^+^ T cell population. Additionally, exosomes from UC-MSCs inhibited CD8^+^ and Th1 cells and reduced their secretion of IFN-γ and TNF-α in a mouse model of contact hypersensitivity (Guo et al., 2019). The exosomes also induced Tregs and promoted their IL-10 secretion. Multiple studies demonstrated that MSCs support the expansion and inhibitory capacity of Tregs and foster their generation from conventional T cells (English et al., 2009; Engela et al., 2013; Khosravi et al., 2017). Involved molecules include HGF, PGE2, TGF-β, and IL-10 (Chen et al., 2020). A recent study confirmed that the promotion of Tregs arises from an epigenetic conversion of conventional T cells to Tregs rather than expansion of natural Tregs (Azevedo et al., 2020). In a murine model of respiratory infection, UC-MSCs were shown to be engulfed by lung phagocytes, which then secreted C-X-C motif ligand (CXCL)9 and CXCL10, leading to the recruitment of particularly suppressive C-X-C motif receptor (CXCR)3^+^ Tregs (Li et al., 2020a). In addition to T helper cells, MSCs also significantly impair the IFN-γ production and cytotoxic activity of CTLs (Maccario et al., 2005; Malcherek et al., 2014).

### B Cells

Activated B lymphocytes produce large amounts of specific antibodies and regulate immune responses by secreting cytokines. Therefore, B cells can crucially contribute to a variety of autoimmune diseases and other antibody-driven pathological mechanisms. MSCs inhibit the proliferation of B cells through an arrest in the G0/G1 phase of the cell cycle (Tabera et al., 2008). Further analyses have shown that MSCs also markedly inhibit the pDC-induced maturation of B cells. Similarly, Magatti et al. (2020) reported a decreased proliferation, maturation, and antibody secretion mediated by MSCs. They also demonstrated that the conditioned medium of MSCs reduces the expression of CD205, CD14, and Toll-like receptor 9 by B cells and that the observed effects are partially mediated by PGE2. A recent study reported that human UC-MSCs impaired the maturation of B cells by secretion of TGF-β (Park et al., 2020). Another important soluble factor is IL1-RA, as MSCs from IL1-RA-deficient mice were unable to inhibit B-cell differentiation (Luz-Crawford et al., 2016). In a mouse model of LPS-induced acute lung injury, MSC treatment blocked the expression of genes involved in chemokine signaling and immunoglobulin expression by B cells (Feng et al., 2020). While multiple studies support the MSC-induced inhibition of B cell antibody secretion, others argue that MSCs actually promote their antibody production as well as survival, proliferation, and differentiation (Traggiai et al., 2008). The effects of activated MSCs on B cells can vary depending on the level of stimulation. Whenever LPS induced a strong IgG production, this effect was reduced by the addition of MSCs, while a lower LPS-induced IgG secretion was associated with an enhancing effect of MSCs (Rasmusson et al., 2007). Several studies support the differential action of MSCs on B cells, depending on their activation status and the environment. For example, IFN-γ-primed pediatric MSCs inhibited naïve, memory, and total B cell proliferation, while unprimed MSCs failed to do so (Palomares Cabeza et al., 2019). Similarly, Luk et al. (2017) demonstrated that AD-MSCs only reduce the proliferation and IgG secretion of B cells upon IFN-γ exposure, while non-stimulated MSCs induced regulatory B cells (Bregs) and IL-10 production. Further analysis revealed that both interactions are dependent on IDO signaling. Interestingly, the separation of B cells and MSCs in a transwell assay inhibited the suppressive properties of MSCs, suggesting that the involved mechanism requires cell contact or at least close proximity. Another study demonstrated that IL-35 plays a crucial role in the induction of IL-10-producing Bregs by MSCs (Cho et al., 2017). Besides the direct secretion of soluble factors, MSCs also mediate their effects on B cells via extracellular vesicles (EVs). For example, EVs from MSCs that were pretreated to mimic inflammatory conditions inhibit the reorganization of the actin cytoskeleton, which is a crucial event during early B cell activation (Adamo et al., 2019). Furthermore, the treatment of B cells with MSC-derived EVs induced the negative modulation of the PI3K-AKT signaling pathway that is involved in cell proliferation and survival. More recently, it has been demonstrated that MSC-EVs block the interaction between follicular T helper cells and germinal B cells, potentially leading to a reduced GvHD score in a mouse model of chronic GvHD (Guo et al., 2020). In contrast, it has also been reported that the effects of AD-MSCs on B cells are mainly mediated by soluble factors and not EVs (Carreras-Planella et al., 2019).

## Conclusion

**FIGURE 1:**
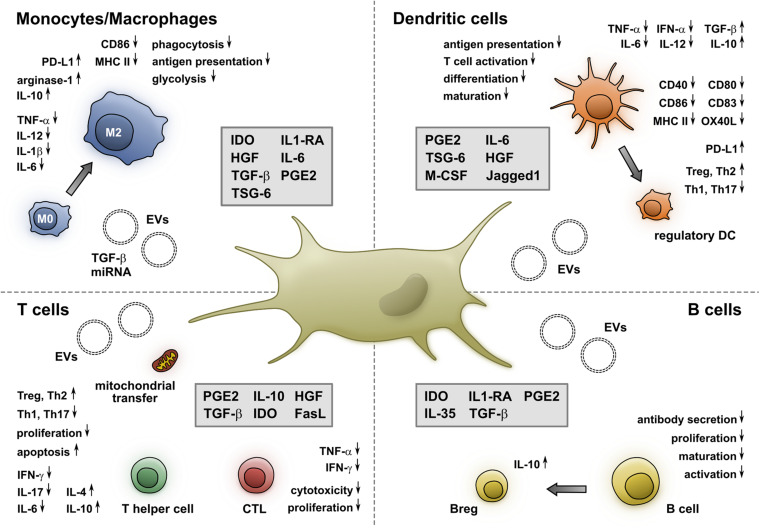
MSCs alter the phenotype and function of monocytes/macrophages, DCs, T- and B-cells. Soluble molecules and EVs released by MSCs induce a shift toward an M2 phenotype, which is accompanied by a reduced secretion of proinflammatory cytokines and an increased production of anti-inflammatory molecules. Furthermore, the expression of CD86 and MHC II as well as their phagocytic and antigen presentation capacity is impaired by MSCs. Additionally, MSCs inhibit the maturation and differentiation of DCs, resulting in a reduced expression of costimulatory molecules, increase the expression of PD-L1, and shift their cytokine profile from proinflammatory toward regulatory. This interaction is mediated by cell-membrane-associated and soluble molecules. MSCs impair both CD4^+^ and CD8^+^ T cells via soluble factors, mitochondrial transfer, and EVs. They also inhibit the generation of Th1 and Th17 cells, while promoting Tregs and Th2 cells. Moreover, they decrease the cytokine secretion and cytotoxicity of CTLs. Furthermore, MSCs hinder the proliferation, maturation, and antibody secretion of B cells and foster the generation of IL-10-producing Bregs.

MSCs display an extraordinary capacity to modulate the phenotype and functional properties of various immune cells (Figure 1). Thus, they excel in inhibiting the maturation and function of DCs and macrophages, while promoting a shift toward regulatory DCs and anti-inflammatory M2 macrophages. They also effectively impair the proliferation and proinflammatory functional properties of B- and T-lymphocytes and induce the generation of Tregs and Bregs. Their immunosuppressive capacities, especially in an inflammatory setting, allow them to efficiently induce clinical responses in patients with immune-mediated disorders (Munneke et al., 2016; Panés et al., 2016; Wang et al., 2016; Bader et al., 2018). Recently, several pre-clinical studies presented promising results in using MSCs or MSC-derived EVs to treat acute lung injury or acute respiratory distress syndrome in Covid-19 patients, and a multitude of clinical studies are currently ongoing (Leng et al., 2020; Moll et al., 2020; Saldanha-Araujo et al., 2020).

However, a significant proportion of patients with immune-mediated disorders fail to respond to MSC-based therapy. The heterogeneity and high plasticity of MSCs entail several challenges and might explain the differences in the clinical outcome. For example, recent evidence suggests that AD-MSCs exhibit a stronger immunosuppressive capacity compared to donor-matched BM-MSCs (Ménard et al., 2020). Besides differing origins, additional differences of studies working with MSCs arise from variations in culture conditions and experimental settings. It has been shown that factors like isolation procedure, seeding density, and media composition alter the phenotype of MSCs, emphasizing the need for standardized protocols (Bara et al., 2014; Czapla et al., 2019; Wang et al., 2020a). Another approach to deal with heterogeneous results in clinical trials is the use of biomarkers to predict the clinical response and pre-select superior donor MSCs and eligible patients. For example, TSG6 expression was shown to be a positive predictor of MSC efficacy in a mouse model of sterile inflammation (Lee et al., 2014). While the ideal biomarker would comprise a univariable marker by which MSCs can be selected prior to therapy, this is unlikely to be achieved due to the extraordinary plasticity of MSCs. Instead, mechanistic and functional evaluation may yield better results, such as the level of cytotoxicity by recipient T and natural killer cells toward MSCs which differed significantly between clinical responders and non-responders in a study with GvHD patients (Galleu et al., 2017). To circumvent the need for cytotoxic actions from recipient cells, the use of apoptotic MSCs proposes another attractive approach and decreases the potential risk of a contribution to tumorigenesis by living MSCs (Weiss and Dahlke, 2019). The cryopreservation required for on demand solutions of viable cells is yet another challenge, as it was shown to influence both phenotypic and functional characteristics of MSCs (Moll et al., 2016). Interestingly, the IFN-γ-mediated licensing of MSCs prior to therapy might not only enhance their immunosuppressive properties but also protect from cryo-induced reduction of viability (Carvalho et al., 2019). Furthermore, cell-free therapies using MSC-derived EVs have gained increasing attention, since they are easier to generate and administer in large quantities, more suitable for long-term storage, and easier to handle in terms of safety and quality control (Phinney and Pittenger, 2017).

Despite these hardships, the capacity to adapt their immunomodulatory effects according to environmental stimuli makes MSCs superior candidates for several therapeutic approaches. Nevertheless, additional research is required to further unveil the underlying molecular mechanisms and determine optimal isolation, cell culture, and cryopreservation conditions to make MSCs meet their expectations in clinical practice.

## Author Contributions

LM and AT drafted the manuscript. MW, MB, RT, MB, FD, RW, and MS reviewed and edited the manuscript. All authors contributed to the article and approved the submitted version.

## Conflict of Interest

The authors declare that the research was conducted in the absence of any commercial or financial relationships that could be construed as a potential conflict of interest.
